# Supplementary material for: Comparative effects of combined aerobic and resistance training versus high-intensity interval training on insulin resistance, glycaemic control, body composition and quality of life in type 2 diabetes: A 12-week randomised controlled trial
Source: PLoS One. 2025 Dec 10;20(12):e0336898. doi: 10.1371/journal.pone.0336898 (PMC12694805; doi:10.1371/journal.pone.0336898)
Supplement: S1 File — (DOCX) [file pone.0336898.s001.docx]

**Appendix 1. Intervention Characteristics by Group (FITT-VP Framework)** [37]

| **Component** | **A+R (Aerobic + Resistance)** | **HIIT (High-Intensity Interval Training)** | **Control** |
| --- | --- | --- | --- |
| **Frequency** | 3–5 sessions/week, 12 weeks | 3–5 sessions/week, 12 weeks | No structured exercise; usual hospital care only |
| **Intensity** | Aerobic: 60–75% HRmaxResistance: 60–70% 1RM | Intervals: 85–90% HRmax (work)Recovery: 50–60% HRmax | – |
| **Time** | 45–60 min/session (20–30 min aerobic + 20–30 min resistance) | 30–40 min/session (incl. 10-min warm-up and 10-min cool-down) | – |
| **Type** | Aerobic: treadmill/cycling Resistance: 6–8 multi-joint exercises (upper & lower limb) | Cycle ergometer or interval walking: 6–10 bouts of 1–3 min work with active recovery | – |
| **Volume** | Aerobic: progressed from 20 to 30 min  Resistance: 2–3 sets × 8–12 reps/exercise | Progressed from 20 min interval phase in week 1–2, to 30 min at week 6, and 45 min by week 12 | – |
| **Progression** | Load/sets/reps increased every 2 weeks based on tolerance and Borg RPE (11–14) | Interval number and duration increased progressively; monitored with Polar HR monitors and Borg RPE (15–17) under physiotherapist supervision | – |

- **A+R Group:** Participants performed combined aerobic and resistance training, beginning with supervised sessions and transitioning to structured home-based sessions after 2 weeks. Exercises targeted all major muscle groups, and resistance loads were increased every 2 weeks based on tolerance.
- **HIIT Group:** The HIIT protocol was adapted from Weston et al. [30] with modifications for safety: 85–90% HRmax (instead of all-out effort), standardised 1-min work/1-min recovery cycles, and extended warm-up/cool-down. Total duration of the interval phase progressed from 20 to 45 minutes over the programme. All sessions were monitored with Polar HR monitors, with weekly physiotherapist supervision.
- Control **Group:** Participants continued usual hospital care and received no structured exercise programme.
- **Adherence:** All participants were advised to complete 3–5 sessions weekly. Adherence was tracked via logbooks and weekly follow-up calls (>70% adherence required). Caregivers assisted illiterate participants.
