# Supplementary material for: Comparative effects of combined aerobic and resistance training versus high-intensity interval training on insulin resistance, glycaemic control, body composition and quality of life in type 2 diabetes: A 12-week randomised controlled trial
Source: PLoS One. 2025 Dec 10;20(12):e0336898. doi: 10.1371/journal.pone.0336898 (PMC12694805; doi:10.1371/journal.pone.0336898)
Supplement: S3 File — (PDF) [file pone.0336898.s003.pdf]

**Comparative Effects of Combined Aerobic and Resistance Training Versus High-Intensity Interval Training on Insulin Resistance, Glycaemic Control, Body Composition and Quality of Life in Type 2 Diabetes: A 12-Week Randomised Controlled Trial**

## INTRODUCTION

Type 2 diabetes mellitus (T2DM) is characterized by chronic hyperglycaemia due to insulin resistance and/or impaired insulin secretion, leading to adverse metabolic, cardiovascular and functional sequelae (DeFronzo et al., 2015; Iaccarino et al., 2021). Exercise is a cornerstone therapy that improves insulin sensitivity, glycaemic control, and cardiovascular risk factors, and is recommended by major guidelines (Colberg et al., 2016; ACSM, 2018). Aerobic training enhances cardiorespiratory fitness and glucose utilization, resistance training increases skeletal muscle mass and glucose uptake, and HIIT offers a time-efficient stimulus producing robust mitochondrial and metabolic adaptations (Boulé et al., 2001; Sigal et al., 2007; Church et al., 2010; Little et al., 2011; Cassidy et al., 2017; Ryan et al., 2020). Despite strong evidence for each modality, direct comparative trials evaluating combined aerobic+resistance (A+R) versus HIIT across metabolic, functional, body-composition and quality-of-life outcomes remain limited, particularly in non-Western settings. This trial will address that gap.

## REVIEW OF LITERATURE

Aerobic and resistance training each improve HbA1c and insulin sensitivity; combined training can yield additive benefits (Sigal et al., 2007; Church et al., 2010; Boulé et al., 2001). Meta-analytic evidence supports structured exercise for lowering HbA1c by ~0.6–0.7% on average (Umpierre et al., 2011) and indicates that resistance training confers additional advantages for glycaemic control and body composition (Strasser and Schobersberger, 2011). HIIT has demonstrated improvements in insulin sensitivity and glucose control with lower time burden (Little et al., 2011; Cassidy et al., 2017). Mechanistically, exercise enhances GLUT4 translocation and mitochondrial biogenesis, underpinning improved insulin action (Hawley and Lessard, 2008; Richter and Hargreaves, 2013). Collectively, this supports testing whether HIIT delivers equal or superior metabolic benefits versus A+R in a pragmatic programme relevant to physiotherapy-led care (Colberg et al., 2016; ACSM, 2018).

## NEED FOR THE STUDY

Given rising T2DM prevalence and variable adherence to lengthy exercise prescriptions, determining the comparative effectiveness and feasibility of A+R vs HIIT is clinically important for tailoring physiotherapist-supervised rehabilitation (Colberg et al., 2016; Umpierre et al., 2011).

AIM

To compare the effectiveness of combined aerobic–resistance training and high-intensity interval training on insulin resistance, glycaemic control, body composition, functional capacity, and quality of life in adults with T2DM.

OBJECTIVES

Assess changes in fasting insulin and HOMA-IR; 2) Evaluate HbA1c and fasting blood glucose; 3) Examine subcutaneous fat, visceral fat and muscle mass; 4) Measure six-minute walk distance (6MWD); 5) Assess WHOQOL-BREF domains.

## CLINICAL SIGNIFICANCE

If effective, structured A+R or HIIT protocols can be embedded into physiotherapy-led diabetes rehabilitation to improve metabolic health, functional capacity and patient-reported outcomes (Colberg et al., 2016; ACSM, 2018).

## METHODOLOGY

**Design:** Three-arm, parallel-group, randomised controlled trial with assessments at baseline and 12 weeks; superiority framework; CONSORT-aligned (Schulz et al., 2010). Setting: Physiotherapy & Diabetes Clinic, tertiary hospital (Ajman, UAE). Duration: 12 months (project lifecycle; intervention 12 weeks).

### Participants

**Inclusion:** Adults 30–65 years; physician-diagnosed T2DM ≥1 year; on stable therapy ≥3 months; cleared for exercise via PAR-Q and AHA/ACSM risk stratification (Thomas et al., 1992; Balady et al., 2007).

**Exclusion:** Type 1 diabetes; uncontrolled hypertension (>180/120 mmHg); significant coronary/respiratory/neurological disease; musculoskeletal limitations; pregnancy; untreated thyroid disorder; inability to consent/participate.

### Sample size

G*Power (repeated-measures, within–between interaction): $f = 0.30$, $\alpha = 0.05$, $1\text{-}\beta = 0.80$, two time-points, $r = 0.50 \rightarrow N = 66$; allowing ~20% attrition → target N = 84 (28/group); we plan N = 90 to ensure power (Faul et al., 2007).

### Randomization, concealment, blinding

Computer-generated blocks (sizes 6–9); allocation concealment via sequentially numbered opaque sealed envelopes (SNOSE); outcome assessors/statistical analysts blinded to group (Schulz et al., 2010).

### Interventions

All participants receive education on hypoglycemia recognition, foot care, and termination criteria; intensity guided by HR and RPE (ACSM, 2018).

### Group A – Combined Aerobic + Resistance (A+R)

Frequency: Aerobic 3 days$\cdot$wk$^{-1}$; Resistance 2 days$\cdot$wk$^{-1}$

Intensity: Aerobic 60–70% HRmax (RPE 11–13); Resistance 60–80% 1RM (RPE 12–15)

Duration: 40–60 min$\cdot$session$^{-1}$

Mode: Brisk walking/cycling; multi-joint exercises for major muscle groups (1–3 sets × 8–12 reps)

Progression: 5–10% every 2 weeks (load or volume) (ACSM, 2018; Sigal et al., 2007).

**Group B – HIIT**

Warm-up/Cool-down: 5 min at ~50% HRmax each

Work-recovery cycles: 1 min at 85–90% HRmax + 1 min active recovery × 10 (20 min core), total 25–35 min

Frequency: 3–5 sessions·$wk^{-1}$

Progression: add cycles or extend work-intervals as tolerated (Little et al., 2011; Cassidy et al., 2017).

**Group C – Control**

Standard diabetes care and lifestyle advice; no structured study exercise during 12 weeks (Umpierre et al., 2011).

Adherence : Exercise logbooks; weekly phone checks; ≥70% session completion defines per-protocol adherence; spot fidelity checks by an independent physiotherapist.

**Outcomes and measurements (baseline; 12 weeks)**

**Metabolic:** Fasting insulin (chemiluminescence immunoassay); fasting glucose; HbA1c (HPLC/immunoassay); HOMA-IR computed from fasting insulin and glucose (Matthews et al., 1985).

**Functional capacity:** 6MWD via ATS protocol (ATS, 2002).

**Body composition:** Bioelectrical impedance analysis (standardised protocol; same device/operator) (Kyle et al., 2004).

**Physical activity:** GPAQ (validity/reliability reported) (Herrmann et al., 2013).

**Quality of life**: WHOQOL-BREF domain scores (validated tool) (WHOQOL Group, 1998). Outcome assessors blinded; device calibration logs maintained.

**Statistical analysis**

Primary analyses via linear mixed-effects models testing group × time for fasting insulin, HOMA-IR, HbA1c; baseline value as covariate (ANCOVA-consistent). Secondary outcomes modelled similarly (6MWD, body composition, GPAQ/sedentary time, WHOQOL domains). Two-sided $\alpha = 0.05$; 95% CIs; standardised mean differences (Cohen's d). Missing data handled with maximum likelihood under MAR; sensitivity multiple imputation if >5% missing (Schulz et al., 2010; Boulé et al., 2001).

**Ethical considerations**

REC approval prior to recruitment; written informed consent; prospective CTRI registration; safety monitoring for hypoglycemia, musculoskeletal events and cardiorespiratory symptoms (Colberg et al., 2016; ACSM, 2018).

## EXPECTED OUTCOMES

We anticipate significant improvements versus control for both interventions. HIIT may yield larger early changes in insulin sensitivity and fasting glucose, whilst A+R may confer broader gains in glycaemic control, body composition and functional capacity (Church et al., 2010; Cassidy et al., 2017; Umpierre et al., 2011).

## ONE-YEAR RESEARCH PLAN SCHEDULE

Q1:Literature synthesis; final protocol; REC/CTRI submissions
Q2:Screening, consent, baseline measures; begin interventions
Q3:Complete interventions; follow-up assessments; data cleaning
Q4: Analyses; write-up; dissemination

## REFERENCES


American College of Sports Medicine (ACSM) (2018) ACSM's guidelines for exercise testing and prescription. 10th edn. Philadelphia: Wolters Kluwer.

American Thoracic Society (ATS) (2002) ATS statement: guidelines for the six-minute walk test. American Journal of Respiratory and Critical Care Medicine, 166(1), pp.111–117.

Balady, G.J. et al. (2007) Recommendations for cardiovascular screening, staffing, and emergency policies at health/fitness facilities. Circulation, 115(17), pp.2358–2368.

Boulé, N.G. et al. (2001) Effects of exercise on glycemic control and body mass in type 2 diabetes mellitus: a meta-analysis. JAMA, 286(10), pp.1218–1227.

Cassidy, S., Thoma, C., Houghton, D. and Trenell, M.I. (2017) High-intensity interval training: a review of its impact on glucose control and cardiometabolic health. Diabetologia, 60(1), pp.7–23.

Church, T.S. et al. (2010) Effects of aerobic training, resistance training, or both on glycemic control in type 2 diabetes. JAMA, 304(20), pp.2253–2262.

Colberg, S.R. et al. (2016) Physical activity/exercise and diabetes: a position statement of the American Diabetes Association. Diabetes Care, 39(11), pp.2065–2079.

DeFronzo, R.A., Ferrannini, E., Zimmet, P. and Alberti, G. (2015) International Textbook of Diabetes Mellitus. 4th edn. Chichester: Wiley-Blackwell.

Faul, F., Erdfelder, E., Lang, A.-G. and Buchner, A. (2007) G*Power 3: a flexible statistical power analysis program. Behavior Research Methods, 39(2), pp.175–191.

Hawley, J.A. and Lessard, S.J. (2008) Exercise training-induced improvements in insulin action. Journal of Applied Physiology, 105(1), pp.274–282.

Herrmann, S.D. et al. (2013) Validity and reliability of the Global Physical Activity Questionnaire. Measurement in Physical Education and Exercise Science, 17(3), pp.221–235.

Iaccarino, G. et al. (2021) Modulation of insulin sensitivity by exercise training: implications for cardiovascular prevention. Journal of Cardiovascular Translational Research, 14(3), pp.256–270.

Kyle, U.G. et al. (2004) Bioelectrical impedance analysis—part I: review of principles and methods. Clinical Nutrition, 23(5), pp.1226–1243.

Little, J.P. et al. (2011) A practical model of low-volume high-intensity interval training improves insulin sensitivity in men with type 2 diabetes. Journal of Applied Physiology, 111(6), pp.1554–1560.

Matthews, D.R. et al. (1985) Homeostasis model assessment: insulin resistance and β-cell function from fasting plasma glucose and insulin. Diabetologia, 28(7), pp.412–419.

Richter, E.A. and Hargreaves, M. (2013) Exercise, GLUT4, and skeletal muscle glucose uptake. Physiological Reviews, 93(3), pp.993–1017.

Schulz, K.F., Altman, D.G. and Moher, D. (2010) CONSORT 2010 statement: updated guidelines for reporting parallel group randomised trials. BMJ, 340, c332.

Sigal, R.J. et al. (2007) Effects of aerobic training, resistance training, or both on glycemic control in type 2 diabetes. Annals of Internal Medicine, 147(6), pp.357–369.

Strasser, B. and Schobersberger, W. (2011) Evidence for resistance training as a treatment therapy in type 2 diabetes. Sports Medicine, 41(4), pp.285–299.

Umpierre, D. et al. (2011) Physical activity advice only or structured exercise training for HbA1c in type 2 diabetes: systematic review and meta-analysis. JAMA, 305(17), pp.1790–1799.

WHOQOL Group (1998) Development of the World Health Organization WHOQOL-BREF. Psychological Medicine, 28(3), pp.551–558.
