# Supplementary material for: Comparative effects of combined aerobic and resistance training versus high-intensity interval training on insulin resistance, glycaemic control, body composition and quality of life in type 2 diabetes: A 12-week randomised controlled trial
Source: PLoS One. 2025 Dec 10;20(12):e0336898. doi: 10.1371/journal.pone.0336898 (PMC12694805; doi:10.1371/journal.pone.0336898)
Supplement: Attachment — Submitted filename: Response to reviewers.pdf [file pone.0336898.s006.pdf]

Response to Reviewers

We sincerely thank the reviewers and editor for their thoughtful and constructive feedback. We have carefully revised the manuscript to address all comments and believe the changes have strengthened the clarity, rigour, and overall impact of the work. Below we provide a detailed, point-by-point response. Each comment is followed by our response and the page/line references where revisions were made in the revised manuscript.

General Editorial Requirements

**Reviewer/Editor Comment**	**Author Response**	**Page/Line Reference**
Trial registration, ethics, data availability, title consistency, style	We confirm that the trial is registered in the Clinical Trial Registry of India (CTRI/2022/04/041762). The full recruitment and follow-up dates (June 2021 to November 2022) have been inserted into the Methods section. We ensured consistency of the title across the manuscript and submission system and relocated the ethics statement exclusively to the Methods. The Data Availability Statement has been revised to confirm that all minimal datasets underlying the findings are included as Supporting Information. These changes bring the manuscript fully in line with PLOS ONE editorial requirements.	Methods – Page 5, paragraph 1; Page 6, paragraph 1; Data Availability section

Reviewer #1

**Reviewer Comment**	**Author Response**	**Page/Line Reference**
Title specificity	We revised the title to include study duration: '... A 12-Week Randomised Controlled Trial.' This addition enhances clarity, improves searchability, and ensures transparency about intervention length.	Title page
Abstract – reporting standards	We revised the Abstract Results to report mean changes with 95% confidence intervals and Cohen’s d effect sizes. This improves clinical interpretability and complies with	Abstract – Page 2, Results section

	reporting standards.	
Introduction – gaps in long-term/diverse studies	We added a dedicated paragraph highlighting the lack of long-term trials and limited inclusion of diverse populations. To strengthen this, we cited Al-mhanna et al. (2024) and Tayebi et al. (2025).	Introduction – Page 4, paragraph 2
Rationale – cost-effectiveness/accessibility	We added justification regarding cost-effectiveness and accessibility of structured exercise, particularly in resource-limited settings, referencing Tayebi et al. (2023, 2024).	Introduction – Page 5, paragraph 1
Novelty – QoL outcomes	We emphasized that this trial integrates clinical and QoL outcomes, a relative novelty compared to many metabolic-focused studies, citing Cobo-Mejía and Castellanos-Vega (2024).	Introduction – Page 5, paragraph 2
Methods – blinding	We clarified that outcome assessors were blinded to group allocation to minimize detection bias.	Methods – Page 7, paragraph 2
Methods – HIIT monitoring	We elaborated on monitoring procedures: Polar HR monitors, HRmax targets, Borg RPE, and physiotherapist supervision. This ensures reproducibility and demonstrates adherence to exercise physiology best practice.	Methods – Page 9, paragraph 3
Results – effect sizes	We added Cohen's d values with 95% CIs for all primary outcomes in Results text and Table 3.	Results – Pages 14–15; Table 3
Discussion – confounders such as diet	We acknowledged that diet was not controlled and cited Al-mhanna et al. (2024) on diet–exercise interactions to contextualize this limitation.	Discussion – Page 21, paragraph 1
Discussion – temper claims of broader benefits	We softened interpretation, noting that while A+R appeared to improve HbA1c and QoL domains, between-group differences were not consistently significant and should be viewed as tendencies.	Discussion – Page 22, paragraph 2
Adherence limitation	We added a note in the Conclusion that findings mainly reflect participants achieving ≥70% adherence. We recommend future studies address	Conclusion – Page 25, paragraph 1

	adherence-enhancing strategies.	
Citations and references	We incorporated recent 2024–2025 literature (e.g., Gajanand et al., Tayebi et al.) to strengthen mechanistic claims and currency.	Throughout Introduction & Discussion
Writing and style	We edited the manuscript for grammar, concision, and consistency (e.g., 'prevention of type 2 diabetes,' consistent 'A+R' terminology).	Throughout manuscript

Reviewer #2

Reviewer Comment	Author Response	Page/Line Reference
Abstract – clarify control group	We specified that A+R and HIIT were compared to a standard-care control group.	Abstract – Page 2
Abstract – clarify primary outcomes	We corrected inconsistencies: fasting insulin, HOMA-IR, and HbA1c are primary outcomes, fasting glucose is secondary.	Abstract & Methods – Page 2; Page 6
Abstract – vague phrasing	We removed 'group × time interactions' and replaced with explicit effect estimates and CIs.	Abstract – Page 2
Abstract – conclusions	We revised conclusions: HIIT yielded greater improvements in fasting glucose and muscle mass, A+R showed broader gains in HbA1c, fat reduction, and QoL domains.	Abstract – Page 2
Introduction – restructure	We restructured into four thematic paragraphs: (1) T2DM burden, (2) exercise modalities, (3) research gaps, (4) study aims and hypotheses.	Introduction – Pages 3–5
Abbreviations	We defined all abbreviations (HbA1c, HOMA-IR, HDL, TGL, T2DM) at first mention and removed unused terms.	Throughout manuscript
Mechanistic rationale	We expanded hypotheses with mechanistic context: HIIT improves insulin sensitivity via GLUT4; A+R enhances fat oxidation and psychosocial benefits.	Introduction – Page 5
Methods – CONSORT compliance	We added explicit statement of compliance with CONSORT 2025 guidelines.	Methods – Page 6, paragraph 1
Define UAE	We clarified 'United Arab Emirates'.	Methods – Page 6
Terminology consistency	We replaced Group A/B/C with A+R, HIIT, Control throughout.	Throughout manuscript
Intervention –	We rewrote intervention description using Frequency,	Methods – Pages

FITT-VP framework	Intensity, Time, Type, Volume, Progression.	7–9
Adverse events placement	We relocated adverse events to Results.	Results – Page 14
Exercise timing	We clarified sessions were mainly late afternoon (Methods) and discussed timing effects on glycaemia (Discussion).	Methods – Page 9; Discussion – Page 21
6MWT phrasing	We corrected to 'distance walked in meters during the six-minute walk test.'	Methods – Page 10
Body composition device	We specified Tanita MC-780MA BIA analyzer.	Methods – Page 10
Terminology – fasting glucose	We standardized to 'fasting glucose' throughout.	Throughout manuscript
Standardization of assessments	We detailed conditions: morning, fasting, no vigorous activity 24h before testing.	Methods – Page 11
Waist/hip measurements	We confirmed ISAK procedures were followed.	Methods – Page 11
Baseline statistical test	We thank the reviewer for this suggestion. However, this is actually a long-standing practice that is not recommended (see: https://doi.org/10.1136/bmj.319.7203.185, http://dx.doi.org/10.31234/osf.io/qftwg, https://doi.org/10.2106/jbjs.21.01166). Any imbalance observed is likely due to randomization, and it does not warrant further analysis (e.g., ANCOVA). Variables we considered important based on background knowledge have already been included as covariates in the analysis. Therefore, we have retained the baseline values in Table 1 but removed the p-values and F-values.	Methods – Page 12
Results – flow and attrition	We expanded CONSORT flow details and noted no attrition.	Results – Page 13
Analysis type	We clarified analyses followed ITT principles.	Methods – Page 12
Medication in Table 1	We added OHA and insulin therapy data.	Results – Table 1, Page 30
Discussion – session timing	We added discussion of time-of-day effects and cited relevant work (PMIDs: 37226675, 37732475).	Discussion – Page 21
Limitations – adherence and generalizability	We expanded Limitations: findings reflect adherent participants, and generalizability may be limited by setting/sample.	Discussion – Page 24
