# Supplementary material for: Comparative effects of combined aerobic and resistance training versus high-intensity interval training on insulin resistance, glycaemic control, body composition and quality of life in type 2 diabetes: A 12-week randomised controlled trial
Source: PLoS One. 2025 Dec 10;20(12):e0336898. doi: 10.1371/journal.pone.0336898 (PMC12694805; doi:10.1371/journal.pone.0336898)
Supplement: Attachment — Submitted filename: Response to reviewer_10.10.25.pdf [file pone.0336898.s008.pdf]

<u>Response to reviewer 3 comments</u>

Comment 1:

Many baseline variables appear to be imbalanced, including age, T2DM duration, FG, PPBS, WHOQOL measures, sedentary time, overweight, eye complications, etc. These imbalances raise concerns about the adequacy of randomisation. Further, the linear mixed model does not specify covariate adjustment. These imbalanced baseline variables should be addressed.

Authors Response:
We thank the reviewer for this valuable observation. We acknowledge that several baseline variables demonstrated numerical differences between groups despite randomisation. Given the modest sample size (n = 30 per group), these imbalances likely occurred by chance. To address this concern and ensure that between-group effects reflect intervention-related changes rather than pre-existing differences, the linear mixed-effects models were re-specified to include key baseline covariates.

The revised model included age, duration of type 2 diabetes mellitus (T2DM), baseline fasting glucose (FG), and baseline WHOQOL-physical domain scores as covariates. This covariate-adjusted model was applied to all primary and secondary outcomes. The Statistical Analysis section has been rewritten accordingly, and the results presented in Table 3 are now explicitly described as covariate-adjusted mean differences (95 % CI).

A statement has also been added to Table 1 legend clarifying that observed baseline imbalances were subsequently adjusted for in the mixed-model analysis. – **Page number 12.**

Comment 2:

In Table 3, add within-group changes. Remove p-values for "interaction" and "between-group" effects, as they are not meaningful for direct between-group comparisons. Cohen's d can be omitted, as mean differences are more clinically interpretable.

Authors Response:
We fully agree with the reviewer's suggestion. Table 3 has been revised to include within-group changes (Post – Pre) for each intervention arm (HIIT, A+R, and Control). All p-values for interaction and between-group effects, as well as Cohen's d values, have been removed. The table now reports covariate-adjusted between-group mean differences (95 % CI) for greater clinical interpretability. Correspondingly, references to effect size calculations have been removed from the Statistical Analysis section. - **Page numbers 15 & 16**

Comment 3:

The assumed effect size (f = 0.30) used in the sample size calculation requires justification.

Authors Response:
We have now added a clear clinical and empirical justification for the assumed moderate effect size (f = 0.30) within the Sample Size Calculation subsection. This assumption was based on prior exercise intervention trials in type 2 diabetes demonstrating moderate changes in HOMA-IR and HbA1c (Bird & Hawley, 2016; Jelleyman et al., 2015; Amaravadi et al., 2024). The text has been revised as follows:

"The assumed moderate effect size (f = 0.30) was based on previous randomised controlled trials in patients with type 2 diabetes reporting moderate improvements in HOMA-IR and HbA1c following structured exercise interventions (Bird & Hawley, 2016; Jelleyman et al., 2015; Amaravadi et al., 2024). This was considered a conservative and clinically meaningful estimate." - **Page number -10**

We thank the reviewer for their thoughtful feedback, which has substantially improved the methodological transparency, statistical rigour, and clinical interpretability of our manuscript.

On behalf of all authors,
Sampath Amaravadi
