# Supplementary material for: Comparative effects of combined aerobic and resistance training versus high-intensity interval training on insulin resistance, glycaemic control, body composition and quality of life in type 2 diabetes: A 12-week randomised controlled trial
Source: PLoS One. 2025 Dec 10;20(12):e0336898. doi: 10.1371/journal.pone.0336898 (PMC12694805; doi:10.1371/journal.pone.0336898)
Supplement: Attachment — Submitted filename: Review_PONE-D-25-40562.docx [file pone.0336898.s004.docx]

**1. Is the Title original, and does it provide a complete and accurate description of the article's content?**

To strengthen it, consider adding specificity on the duration (e.g., "12-Week") for better searchability.

**2. Does the Abstract provide a complete and accurate description of the article's content?**

For improvement, include effect sizes or confidence intervals in the results section to align with modern reporting standards seen in recent meta-analyses, providing readers with a quicker grasp of clinical significance.

**3. Are study problems and gaps clearly stated based on the recent studies in the introduction section?**

To enhance, explicitly discuss gaps in long-term or diverse population studies:

- Badri Al-mhanna S, Wan Ghazali W S, Batrakoulis A, Alkhamees N H, Drenowatz C, Mohamed M, et al . The Impact of Various Types of Exercise on Lipid Metabolism in Patients with Type 2 Diabetes and Concurrent Overweight/Obesity: A Narrative Review. Ann Appl Sport Sci 2024; 12. [10.61186/aassjournal.1324](http://dx.doi.org/10.61186/aassjournal.1324)
- Tayebi, S.M., Bagherian, P., Bassami, M., Basereh, A. and Ahmadabadi, S. (2025), Impact of a 12-week High-Intensity Interval Training With Spirulina Supplementation on Insulin Resistance-Mediated by Apo-A, -B, and -J in Men With Obesity. Eur J Sport Sci, 25: e12285. <https://doi.org/10.1002/ejsc.12285>

**4. Is the rationale/justification for conducting the study clearly stated in the introduction section?**

Strengthen by adding a sentence on potential cost-effectiveness or accessibility:

- Tayebi S M, Saeidi A, Shahghasi R, Golmohammadi M. The Eight-Week Circuit Resistance Training Decreased the Serum Levels of WISP-1 and WISP-2 in Individuals with Type 2 Diabetes. Ann Appl Sport Sci 2023; 11 (4). [10.61186/aassjournal.1290](http://dx.doi.org/10.61186/aassjournal.1290)
- Tayebi, S. M., Motaghinasab, S., Eslami, R., Ahmadabadi, S., Basereh, A., & Jamhiri, I. (2024). Impact of 8-week cold-and warm water swimming training combined with cinnamon consumption on serum METRNL, HDAC5, and insulin resistance levels in diabetic male rats. *Heliyon*, *10*(8). [10.1016/j.heliyon.2024.e29742](https://doi.org/10.1016/j.heliyon.2024.e29742)

**5. Are the study aims and novelty clearly stated in the introduction section, and are they logical?**

To bolster novelty, contrast with prior studies focusing only on metabolic markers, emphasizing the inclusion of QoL:

- Cobo-Mejía E A, Castellanos-Vega R D P. The Effects of Physical Activity on Glycated Hemoglobin and Quality of Life in Adults with Diabetes Mellitus: A Systematic Review of the Literature and Meta-Analysis. Ann Appl Sport Sci 2024; 12 (4). [10.61186/aassjournal.1420](http://dx.doi.org/10.61186/aassjournal.1420)
- Tayebi S M, Eslami R, Iranshad I, Golmohammadi M. The Effect of Eight Weeks of Circuit Resistance Training on Serum Levels of GPR119 and β-Arrestin1 in Individuals with Type 2 Diabetes. Ann Appl Sport Sci 2023; 11 (3). [10.61186/aassjournal.1283](http://dx.doi.org/10.61186/aassjournal.1283)

**6. In the materials and methods section, is the study design robust and appropriate to the stated aim?**

Suggest including blinding details (e.g., assessor blinding) to mitigate bias.

**7. Are the methods described (like Participants/Patients/Animals, Exercise Training Protocol/ Study Tools and Assessments, Data Collection Protocol, Statistical Analysis, etc.) in sufficient detail and standard methods to reproduce the experiment?**

For enhancement, provide more specifics on HIIT intensity monitoring (e.g., heart rate devices) and progression criteria.

**8. In the results section of the full text, are the contents reported in a manner that is consistent with reporting guidelines for the article/study type?**

To improve, add effect sizes (Cohen's d) for all outcomes, as done in the primary analysis description, to better quantify clinical relevance.

**9. Is the discussion section critical and comprehensive with a robust, valid, and reliable data interpretation (analyze the proper findings presentation, Logical Comparison with the Related Literature, Interpretation of the Findings, and the Mechanisms of these Findings)?**

Strengthen by discussing potential confounders (e.g., diet) and integrating more 2024-2025 Stidies. I sugessted some updated and related references above. Use and cite them.

**10. Are the conclusions drawn supported by the data, or need to be adjusted with new revisions?**

No major revisions needed, but temper claims on "broader benefits" by noting non-significant between-group differences in some outcomes.

**11. Are the applicable remarks prescribed based on the exact findings of this study, or do they need to be adjusted with new revisions?**

Remarks on personalized prescriptions are directly based on findings (e.g., modality-specific strengths). Implications for clinicians are practical and evidence-based. Adjust slightly to emphasize adherence limitations, as results apply to compliant patients, and suggest hybrid models for future.

**12. Are statements supported appropriately by citations?**

Add citations for all mechanistic claims to enhance rigor. I sugessted some updated and related references above. Use and cite them.

**13. Are the references appropriate in number and up-to-date?**

36 references are appropriate for an RCT; many are recent (2020-2025, e.g., IDF 2025, Gajanand 2024), aligning with knowledge updates. Include more 2024-2025 meta-analyses for HIIT vs. combined training to ensure currency. I sugessted some updated and related references above. Use and cite them.

**14. Are figures and tables well-constructed and of sufficiently high resolution (i.e., not blurry)?**

All seems ok.

**15. Are figures and tables well-annotated and easy to read and interpret?**

All seems ok.

**16. Is the writing clear, concise, and logical?**

Writing is generally clear and logical, with structured flow. Some sentences are wordy (e.g., introduction repetitions on exercise benefits). Concise overall, but edit for grammar (e.g., "preventing of type-2" to "prevention of type 2"). This would make it more engaging and professional.

**17. Does the writing impede scientific meaning or cause confusion?**

No major impediments, but minor inconsistencies (e.g., "A+R" vs. "combined aerobic and resistance") and awkward phrasing (e.g., "normalizes in the prevention") could confuse. Revise for precision to avoid ambiguity, ensuring scientific accuracy.
