# Supplementary material for: Comparative effects of combined aerobic and resistance training versus high-intensity interval training on insulin resistance, glycaemic control, body composition and quality of life in type 2 diabetes: A 12-week randomised controlled trial
Source: PLoS One. 2025 Dec 10;20(12):e0336898. doi: 10.1371/journal.pone.0336898 (PMC12694805; doi:10.1371/journal.pone.0336898)
Supplement: Attachment — Submitted filename: Response to reviewer.docx [file pone.0336898.s009.docx]

# **Response to Reviewer #3**

## **Reviewer #3 Comment:**

P-values should be adjusted to account for the large number of primary and secondary outcomes analyzed compared to the small sample size.

## **Authors’ Response:**

We sincerely thank the reviewer for this valuable observation and agree that adjustment for multiple comparisons is an important consideration in studies with several outcomes and a modest sample size.

We would like to clarify that baseline P-values were not presented in the first place because this practice is no longer recommended in randomized controlled trials. As previously discussed, this is a long-standing practice that has been widely discouraged in the literature (see:<https://doi.org/10.1136/bmj.319.7203.185>, <http://dx.doi.org/10.31234/osf.io/qftwg>, <https://doi.org/10.2106/jbjs.21.01166>). Any imbalance observed at baseline is likely due to randomization and does not warrant inferential testing (e.g., ANCOVA). Variables that are clinically or theoretically important were already included as covariates in the mixed-model analysis to account for any potential baseline variation.

However, we fully respect the reviewer’s concern regarding the potential for inflated Type I errors due to multiple comparisons. To address this, we have re-evaluated the analyses and adjusted all P-values for multiple testing using both the Benjamini–Hochberg False Discovery Rate (FDR, q = 0.05) and Bonferroni correction (m = 23 tests). The adjusted P-values are now reported in Table 1 and Table 3 to ensure full transparency and statistical robustness.

The Statistical Analysis section has been revised to include the following statement:
“Given the relatively small sample size and the number of primary and secondary outcomes, P-values were adjusted using both the Benjamini–Hochberg False Discovery Rate (FDR, q = 0.05) and Bonferroni correction (m = 23 tests; adjusted α = 0.002). Adjusted P-values are presented in the tables to account for multiple comparisons and ensure alignment with CONSORT 2025 recommendations.”

These revisions did not materially change the overall interpretation of the findings, as the principal outcomes and direction of effects remained consistent with the original analysis. We believe that these revisions further strengthen the methodological rigour, transparency, and reproducibility of the study, while respectfully addressing the reviewer’s concern.

## Changes Made:

• Abstract – results section (Page 2-3) & Statistical Analysis section revised (Page 10).

• Adjusted P-values added to Table 1&3 (Pages 12-13).

• Results updated to reflect FDR and Bonferroni-adjusted P-values. (Page 11)

On behalf of all authors,
Sampath Amaravadi
