# Supplementary material for: Comparative effects of combined aerobic and resistance training versus high-intensity interval training on insulin resistance, glycaemic control, body composition and quality of life in type 2 diabetes: A 12-week randomised controlled trial
Source: PLoS One. 2025 Dec 10;20(12):e0336898. doi: 10.1371/journal.pone.0336898 (PMC12694805; doi:10.1371/journal.pone.0336898)
Supplement: Attachment — Submitted filename: Response to reviewer comments.pdf [file pone.0336898.s007.pdf]

# RESPONSE TO REVIEWERS

Dear Editor,

We thank the reviewers for their detailed feedback and for recognizing the improvements made in the revised manuscript. We are pleased that Reviewer #1 finds the work suitable for publication.

We have carefully addressed the additional clarifications requested by Reviewer #2. Below, we provide a point-by-point response, with tracked changes references to the revised manuscript.

**Reviewer #2**

**Comment 1: Results section**

"Please provide mean differences between groups for all outcomes, as this aligns with the study's primary research question of superiority testing. Reporting within-group changes is unnecessary and may be misleading; it is sufficient to include these in the tables."

**Response:**
We have revised the Results to focus exclusively on between-group mean differences (MDs) with 95% CIs, consistent with superiority testing. All within-group changes have been removed from the narrative and retained only on tables.

**Change made:** For example, the Results now read (Page 12, Lines 310-353): "The HIIT group demonstrated a greater reduction in fasting glucose compared with control (MD −29.1 mg/dL; 95% CI −41.2 to −17.0), and the A+R group also improved relative to control (MD −20.6 mg/dL; 95% CI −31.0 to −10.2) (Table 3)."

**Other outcomes:** HbA1c, fasting insulin, HOMA-IR, body composition, functional capacity, and quality of life have all been updated in the same way.

**Comment 2: Abstract**

"The same principle should be applied in the abstract, ensuring consistency with the research question."

**Response:**
We have revised the Abstract to report intervention vs control MDs with 95% CIs for the primary and secondary outcomes, ensuring consistency with the Results.

**Change made:** Abstract, Results section (Page 2-3, Lines 36–49): "Compared with control, the HIIT group reduced fasting glucose (MD −29.1 mg/dL; 95% CI −41.2 to −17.0) and A+R also improved (MD −20.6 mg/dL; 95% CI −31.0 to −10.2). HbA1c was lower in HIIT (MD −3.35%; 95% CI −4.11 to −2.58) and A+R (MD −3.33%; 95% CI −4.03 to −2.62) compared with control…"

**Comment 3: Baseline characteristics**

"My main suggestion was to report the total sample size available for each outcome at baseline … Based on the flowchart, it appears that no missing values are present, so this is not

needed. … I suggest removing this information from the tables and manuscript to avoid confusion.”

**Response:**
We confirm that all participants completed baseline assessments with no missing data, as shown in the flow diagram. Following the reviewer’s advice:

**Change made in Tables 1–2:** All baseline statistical tests (ANOVA/$\chi^2$) and p-values have been removed. Only descriptive values (mean ± SD or n [%]) are presented. (Page 11-12, Table 1&2).

**Change made in Methods (Statistics):** Clarified that baseline characteristics are presented descriptively, with no hypothesis testing (Page 10, Lines 259–261).

We believe these revisions fully address Reviewer #2’s comments. All requested clarifications have been incorporated, with tracked-changes visible in the revised manuscript (clean and marked versions uploaded). We hope the manuscript is now acceptable for publication.

With thanks,

Sincerely,

Sampath
